# Nerve Transfers to Restore upper Extremity Function: A Paradigm Shift

**DOI:** 10.3389/fneur.2014.00040

**Published:** 2014-03-31

**Authors:** Amy M. Moore

**Affiliations:** ^1^Plastic and Reconstructive Surgery, Washington University School of Medicine, St. Louis, MO, USA

**Keywords:** nerve transfer, brachial plexus injury, peripheral nerve injury, surgical procedures, operative, upper extremity, hand function, upper extremity function

Brachial plexus and peripheral nerve injuries lead to significant upper extremity dysfunction and disability. Traditionally, both have been treated with nerve grafting when a tensionless, end-to-end repair is not feasible. Despite our best efforts, functional outcomes of this procedure are less than ideal due to the long distances that the axons must regenerate to reach their end organs. Over the past 20 years our understanding of nerve anatomy, topography, and regeneration has improved and the surgical technique of nerve transfers has been developed. Due to improved functional outcomes, decreased morbidity, and surgical time, we are now experiencing a paradigm shift in the treatment of brachial plexus and peripheral nerve injuries from nerve grafting to nerve transfers (1, 2).

Motor function after nerve injury is dependent on both time to reinnervation and the number of motor axons reinnervating the target muscle (3). Nerve transfers capitalize on these two factors and are the reason for their clinical success. Nerve transfers, by definition, involve coapting a healthy, expendable donor nerve or fascicle to a denervated recipient nerve to restore function to the recipient end-organ (skin for sensation or muscle for motor function). They can be performed closer to the recipient target allowing for earlier reinnervation of the muscle and quicker return of function. Further advantages include that nerve transfers are performed outside the zone of injury and scarred field, can be performed on patients with delayed presentation, and can avoid interpositional nerve grafting, which leads to increased numbers of regenerating nerve fibers making it to the target organ (3).

The ideal timing of nerve transfers has not yet been established, but reinnervation of the muscle by 12–18 months after injury is a common goal. Indications are evolving and currently include patients with proximal nerve root avulsions, high level peripheral nerve injuries, large neuromas-in-continuity, and/or multi-level nerve injuries. In our group, we use nerve transfers to treat most brachial plexus injuries (avulsions or not) and peripheral nerve injuries in upper arm or proximal forearm. We usually reserve nerve grafting for nerve injuries in the distal forearm or hand because the regenerative distances and time to reinnervation of the muscle are short. At these distal injuries, functional outcomes with grafting are similar to those seen with nerve transfers and donor site morbidity from a nerve transfer is avoided.

In brachial plexus injuries, a hierarchy of return of function exists with efforts directed to restoring elbow flexion first, followed by shoulder function, then hand function. For upper trunk injuries, multiple combinations of nerve transfers have been described. The double fascicular nerve transfer is the most common nerve transfer performed to return elbow flexion. This transfer involves coapting redundant nerve fascicles from the median and ulnar nerves to the biceps brachii and brachialis branches of the musculocutaneous nerve. Many have reported their experience with this transfer and patients have achieved at least Medical Research Council (MRC) strength of 3 with most achieving grade 4 or greater without evidence of donor site morbidity (4–6). For restoration of shoulder function transfers of the spinal accessory nerve to the suprascapular nerve and a branch of the triceps to the axillary nerve are most commonly performed. Thoracodorsal nerve and intercostal nerves transferred to the long thoracic nerve are also common to restore scapular stability provided by the serratus anterior muscle. Restoration of shoulder abduction and external rotation has been successfully reported with these nerve transfers (7, 8). In lower plexus injuries, the brachialis branch of the musculocutaneous nerve can be transferred with encouraging results to the anterior interosseous nerve to restore prehension. Previously, these lower plexus injuries were treated with free functional muscle transfers given the great regenerative distance from the brachial plexus to the forearm musculature. However, free functional muscle transfers are associated with increased morbidity, operative time, and lengthy hospital stays. The brachialis to anterior interosseous nerve transfer avoids these drawbacks and establishes a platform for restoring function to the hand.

In addition to their use for brachial plexus injuries, nerve transfers to restore hand function following peripheral nerve injuries are also gaining momentum. New transfers continue to be developed as our understanding of nerve topography grows. Ulnar nerve injuries result in loss of power grip, pinch strength, and hand dexterity. The pronator quadratus branch of the anterior interosseous nerve can be transferred to the motor component of the ulnar nerve distally in the forearm to reinnervate the intrinsic muscles of the hand (9). It was originally described as an end-to-end coaptation if no regeneration of the ulnar nerve is expected, but recently Mackinnon and colleagues have shown efficacy of an end-to-side “supercharge” coaptation enabling proximal regeneration of the ulnar nerve to proceed as well (10). Upper extremity trauma frequently results in radial nerve injuries impairing both wrist and finger extension. Although tendon transfers are functional for patients with radial nerve palsies, nerve transfers from the median to radial nerves allow for independent thumb and finger extension (11). To restore median nerve function, transfer of branches of the radial nerve, the brachialis branch, and branches of the ulnar nerve have been described with good outcomes (12). Focusing on synergism and redundancy of function has led to the success of these transfers.

An exciting application of nerve transfers is in the field of spinal cord injury (SCI). Drs. Susan Mackinnon and Ida Fox at Washington University in St. Louis, MO, USA are leading developers in the use of nerve transfers to restore upper extremity function in patients with cervical SCI. These transfers are being developed to increase volitional control and improve independence. Unlike brachial plexus or peripheral nerve injuries, SCI patients have intact lower motoneurons below the level of injury and thus, the motoneuron – peripheral nerve – muscle end-organ connection remains intact. For this reason, the muscle is “preserved” and nerve transfers in SCI patients can be performed without the time sensitivity found with a peripheral nerve injury. Specific transfers for SCI include transfer of the brachialis branch of the musculocutaneous nerve to the anterior interosseous nerve to improve prehension and transfer of the deltoid nerve branches to the triceps branches to improve elbow extension. Evaluation and collaboration among the physiatrists, therapists, and surgeon are critical to identifying ideal candidates, developing operative plans, and ultimately achieving success with nerve transfers in this patient population.

In conclusion, nerve transfers are an essential tool for the peripheral nerve surgeon to improve upper extremity function after nerve injury. I would argue that nerve transfers are the preferred treatment for high peripheral nerve injuries and for most patterns of brachial plexus injury. In addition, they will likely play an increasing role in managing SCI patients. Return of earlier, more effective upper extremity function supports the importance of this surgical technique. As we critically analyze and report our outcomes with nerve transfers, further indications and expectations of return of function will be elucidated. The paradigm shift; however, is happening now. Nerve transfers viewed as “standard of care” may not be far away. Currently, they certainly hold great promise and should be considered in restoring upper extremity function in patients with devastating nerve injuries.

## References

[1] Garg R Merrell GA Hillstrom HJ Wolfe SW Comparison of nerve transfers and nerve grafting for traumatic upper plexus palsy: a systematic review and analysis. J Bone Joint Surg Am (2011) 93:819–29 10.2106/JBJS.I.01602 21543672

[2] Yang LJ Chang KW Chung KC A systematic review of nerve transfer and nerve repair for the treatment of adult upper brachial plexus injury. Neurosurgery (2012) 71:417–29 discussion 429, 10.1227/NEU.0b013e318257be98 22811085

[3] Tung TH Mackinnon SE Nerve transfers: indications, techniques, and outcomes. J Hand Surg Am (2010) 35:332–41 10.1016/j.jhsa.2009.12.002 20141906

[4] Belzberg AJ Dorsi MJ Storm PB Moriarity JL Surgical repair of brachial plexus injury: a multinational survey of experienced peripheral nerve surgeons. J Neurosurg (2004) 101:365–76 10.3171/jns.2004.101.3.0365 15352592

[5] Teboul F Kakkar R Ameur N Beaulieu JY Oberlin C Transfer of fascicles from the ulnar nerve to the nerve to the biceps in the treatment of upper brachial plexus palsy. J Bone Joint Surg Am (2004) 86-A:1485–90 15252097 10.2106/00004623-200407000-00018

[6] Ray WZ Pet MA Yee A Mackinnon SE Double fascicular nerve transfer to the biceps and brachialis muscles after brachial plexus injury: clinical outcomes in a series of 29 cases. J Neurosurg (2011) 114:1520–8 10.3171/2011.1.JNS10810 21351836

[7] Leechavengvongs S Witoonchart K Uerpairojkit C Thuvasethakul P Nerve transfer to deltoid muscle using the nerve to the long head of the triceps, part II: a report of 7 cases. J Hand Surg Am (2003) 28:633–8 10.1016/S0363-5023(03)00199-0 12877852

[8] Bertelli JA Ghizoni MF Reconstruction of C5 and C6 brachial plexus avulsion injury by multiple nerve transfers: spinal accessory to suprascapular, ulnar fascicles to biceps branch, and triceps long or lateral head branch to axillary nerve. J Hand Surg Am (2004) 29:131–9 10.1016/j.jhsa.2003.10.013 14751116

[9] Wang Y Zhu S Transfer of a branch of the anterior interosseus nerve to the motor branch of the median nerve and ulnar nerve. Chin Med J (Engl) (1997) 110:216–9 9594344

[10] Barbour J Yee A Kahn LC Mackinnon SE Supercharged end-to-side anterior interosseous to ulnar motor nerve transfer for intrinsic musculature reinnervation. J Hand Surg Am (2012) 37:2150–9 10.1016/j.jhsa.2012.07.022 23021177

[11] Ray WZ Mackinnon SE Clinical outcomes following median to radial nerve transfers. J Hand Surg Am (2011) 36:201–8 10.1016/j.jhsa.2010.09.034 21168979 PMC3031762

[12] Brown JM Mackinnon SE Nerve transfers in the forearm and hand. Hand Clin (2008) 24:319–40 10.1016/j.hcl.2008.08.002 18928884

